# Transgenic animal models for the production of human immunocompounds in milk to prevent diarrhea, malnourishment and child mortality: perspectives for the Brazilian Semi-Arid region

**DOI:** 10.1186/1753-6561-8-S4-O30

**Published:** 2014-10-01

**Authors:** Luciana Bertolini, Marcelo Bertolini, James Murray, Elizabteh Maga

**Affiliations:** 1 University of Fortaleza, UNIFOR, Fortaleza, Brazil; 2 University of California-DAVIS, Davis, CA, USA

## 

Diarrhea is one of the most significant issues for global health in children under the age of five. Close to 60% of mortality in this risk group is caused by pneumonia, diarrhea or measles, which is usually associated with malnutrition. In Brazil, this scenario is no different, with the Northeast semi-arid region having alarming rates of child mortality, almost twice that of the current national mortality rate, with some cities being three times higher, placing child mortality rates in the Northeast of Brazil among those of the most problematic in the world. The oral rehydration solution, introduced by the World Health Organization (WHO), is seen as one of the major medical advances in the past 50 years, and is believed to save the lives of 1 to 2 million children each year. However, the oral rehydration solution simply restores body fluid normality, treating the consequences and not the causes of diarrheas. Other means to effectively address worldwide diarrheic ailments still need to be devised or improved, focusing more on preventive rather than curative approaches, to prevent or shorten the course of disease and to minimize recurrences. A great example is breast-feeding. A series of studies documented the reduction of diarrhea episodes in breast-fed children and, also, noted a faster recovery time following an infection in these children. The positive benefit of breast milk is attributed to the antimicrobial actions of human milk proteins, such as lysozyme and lactoferrin that can enhance intestinal and systemic immunological functions. Unfortunately, breast-feeding is not always an option, especially for toddlers, which is usually aggravated by undernourishment and low standard of living, reinforcing the need for alternative strategies. Consequently, to successfully combat childhood undernourishment, morbidity and mortality in the Northeast region of Brazil, the investment in approaches and models that are more suitable to the local semi-arid adapted agriculture-base is required.

The goat is an important economic asset to the Northeast region, providing milk, meat and leather, with the number of goats in the Northeast region representing more than 90% of the Brazilian herd. Historically, goats have been used as a model in biotechnological studies involving the expression of specific proteins in the milk of lactating animals. In this way, the use of goats expressing recombinant human lysozyme (rhLZ) and lactoferrin (rhLF) in milk is a proven reality that can be employed for the reduction of malnutrition, infectious diseases and diarrhea in children in the Brazilian semi-arid region with an extraordinary potential for success. Due to the protective nature of human milk imparted, in part, by the presence of lysozyme and lactoferrin, the generation of transgenic lines for the production of these important antimicrobial compounds in the milk of the local goat population is a unique approach for delivering the potential protective benefits of human milk. In addition, the use of rhLZ and rhLF transgenic animals can be used as a research tool for studying and elucidating the individual roles of these antimicrobial proteins in gut development and their synergistic *in vivo *effects.

The proposed research was designed with the rational that the transgenic approach can be used to modify the milk composition of dairy animals to supply milk-borne human immunocompounds (rhLZ, rhLF) and nutrients to children in a continuous manner. Therefore when consumed, this milk would impart enhanced immune capabilities that would work to benefit the growth and health of the young and also help in the management of diarrheal diseases. Genetically modified animal models for the mammary gland expression of rhLZ, and more recently rhLF, have been systematically studied at the University of California at Davis (UC Davis) over the past 20 years, with the milk used for extensive *in vitro *and *in vivo *studies. Lines of transgenic mice and goats with genetically altered milk for the expression of rhLZ were created at UC Davis. More recently, investigators at UC Davis have also initiated parallel and similar studies using the milk of cows expressing rhLF produced and licensed by Pharming Inc. Early in this century, a research consortium was created between researchers at UC Davis and investigators in Brazil at the University of Fortaleza (UNIFOR) and Federal University of Ceará (UFC), in the Brazilian northeast region, with the long-term goal of improving child health by focusing on means to reduce child mortality and morbidity caused by gastrointestinal ailments (diarrhea) and malnutrition and also aiming to promote the development of the Northeast semi-arid region of Brazil. In 2012, a transgenic goat line producing rhLZ was produced in Brazil at UNIFOR, with animals and milk being under investigation focusing on similar validating *in vitro *and *in vivo *studies as performed at UC Davis, translating results and adapting the studies to the conditions of the Brazilian northeast region.

The concept of producing a milk with enhanced antimicrobial properties was first tested in mouse models in the early 1990s where milk from transgenic mice expressing rhLZ was bacteriostatic against cold-spoilage organisms and a mastitis-causing strain of *Staphylococcus aureus*, with results in mice suggesting that rhLZ into the milk was effective in inhibiting bacterial growth. The Artemis line of rhLZ transgenic goats was then established in 1999 at UC Davis and since then, extensive and multiple risk assessment studies carried out on the transgenic line have shown no negative impact of the presence or expression of the lysozyme transgene on animal well-being, health, growth, reproduction and milk production and composition. Furthermore, using microbial and metabolite profiling techniques to investigate any effects on the physiology of the host (lactating goats) at the whole animal level, no unintended effects of transgene expression were found. Milk from rhLZ transgenic goats possesses enhanced antimicrobial activity over milk from non-transgenic control goats as it significantly slowed the growth of pathogenic bacteria *in vitro*. Milk from rhLZ transgenic goats also has a longer shelf-life with fewer bacteria growing in milk of transgenic animals and milk survived at room temperature for longer periods than control milk before bacterial growth occurred. Milk from transgenic animals also had a lower somatic cell count, a measure commonly used to monitor the state of infection of the mammary gland from lactation to lactation, thereby indicating modulation by rhLZ resulting in a healthier udder. Upon consumption by both ruminant and non-ruminant animal models, pasteurized milk from rhLZ transgenic goats was capable of modulating intestinal bacteria. Weaning pigs serving as a human-relevant animal model receiving milk from rhLZ transgenic animals for 16 days had significantly lower numbers of coliforms and *E. coli *in their intestine than did pigs fed milk from non-transgenic control animals. Compared to control milk-fed pigs, the microbiota of rhLZ-fed pigs more closely resembled that of human infants being breast-fed with the enrichment of *Bifidobacteriacea *and *Lactobacillacea*, both biomarkers of increased gut and host health. These beneficial changes were accompanied by the reduction of clostridia and *Streptococcaceae*, which are components of the fecal microbiota of infants fed formula that lacks lysozyme as well as decreased levels of disease-related bacteria such as *Mycobacteriaceae *and *Campylobacterales*. This study demonstrated that consumption of lysozyme-rich milk resulted in beneficial microbe enrichment and detrimental microbe reduction in the gut microbiome community. Altogether, these data indicate that rhLZ expressed in the milk of dairy animals can indeed be biologically active in the intestine and modulate gut microbiota, much like human milk. Bacterial challenge work was also conducted in the pig as a human-relevant medical model. To determine if lysozyme-rich milk could influence the clinical state of the animal, rhLZ or control milk was fed to six-week old pigs that had active diarrhea as induced by administration of a porcine-specific enterotoxigenic *E. coli *(ETEC). Pasteurized milk from rhLZ transgenic or control goats was then given for 48 h. Fecal scores and clinical status were recorded throughout the trial. Pigs receiving milk from rhLZ transgenic animals had significantly improved fecal scores (less diarrhea) and by the end of the trial suffered from less dehydration and cleared the infection faster than pigs receiving control milk. These results demonstrate that rhLZ alone in milk is enough to have a clinical effect on the symptoms of diarrhea in the pig model. Finally, two animal models for malnourishment were recently used to evaluate the effect of dietary supplementation with rhLZ goat milk or rhLF cow milk. Using a rat malnourished model, the daily supplementation with rhLZ goat or control milk for a period of two weeks resulted in the reestablishment of the body weight and gut morphology in malnourished rats receiving the transgenic milk to values similar to full-fed control animals, demonstrating a positive effect in the physiological recovery from malnourishment. Using a pig model for malnourishment, the supplementation of the diet with control or rhLF milk significantly improved intestinal morphology and permeability, promoting gut recovery and demonstrating that the administration of both control and rhLF milk was beneficial in the recovery of the gastrointestinal tract. Our intention is that such milk from transgenic animals can protect and benefit malnourished children around the world.

In summary, the data obtained thus far has indicated no detrimental effects of the transgenic rhLZ goat or rhLF cattle lines on milk properties and processing, and on general well-being and animal health. In addition, milk from lysozyme transgenic goats and lactoferrin transgenic cows have shown positive impacts on gut microbiota and morphology, significantly assisting in mitigating intestinal damage caused by malnutrition, and helped to resolve *E. coli*-induced diarrhea and clinical signs more quickly than control milk. This work has progressed from testing the use of transgenics to modify the properties of milk in a mouse model to the testing of the efficacy of the rhLZ and rhLF milk to act at the level of the intestine in the pig and rat models of human health. Work is now focusing on the mechanism of action of rhLZ and rhLF in milk, also moving to the rat model for additional pharmacological and toxicity testing as one of the last steps before human clinical trials. The ongoing studies proposed by the research consortium represents an important step towards the ultimate goal of using genetic engineering to improve the health and quality of animal-based food products designed for human consumption. The use of milk with rhLZ and rhLF represents a viable approach to reduce morbidity and mortality due to diarrhea and malnutrition, thereby resulting in the potential to promote the development and improvement in the quality of life in the semi-arid region. The availability of livestock milk with human milk properties appears to be a potential food source to enhance and prolong the protective benefits of human milk by promoting disease reduction and growth enhancement. In fact, the mammary gland transgene expression systems for the production of functional proteins in the milk of animals have already been proven as a viable technological alternative to aid in the resolution of problems of the modern world as a means of human pharmaceutical production. It is expected and anticipated that such research efforts will lead to milk that serves as food to nourish and to protect infants and children against diarrhea illnesses in unfavorable regions of the world, also providing an enhanced oral rehydration solution for diarrheal infections that each year, according to the WHO, are responsible for the death of close to a million children worldwide.

